# LINC00963 promotes the malignancy and metastasis of lung adenocarcinoma by stabilizing Zeb1 and exosomes-induced M2 macrophage polarization

**DOI:** 10.1186/s10020-022-00598-y

**Published:** 2023-01-05

**Authors:** Ronghang Hu, Baobin Xu, Jiajun Ma, Linfeng Li, Liming Zhang, Li Wang, Jiebo Zhu, Tao Guo, Heng Zhang, Shaoqiang Wang

**Affiliations:** 1 Department of Thoracic Surgery, Affiliated Hospital of Jining Medical University, Jining Medical University, Jining, 272029 Shandong People’s Republic of China; 2 Department of Thoracic Surgery, Xiangya Hospital, Central South University, No. 87, Xiangya Road, Changsha, 410008 Hunan People’s Republic of China; 3 Department of Thoracic Surgery, The Second Xiangya Hospital of Central South University, Changsha, 410011 Hunan People’s Republic of China; 4 Xiangya Lung Cancer Center, Xiangya Hospital, Central South University, Changsha, 410008 Hunan People’s Republic of China; 5 National Clinical Research Center for Geriatric Disorders (Xiangya Hospital), Changsha, 410008 Hunan People’s Republic of China; 6 Department of Thoracic Surgery, Weifang People’s Hospital, Weifang Medical University, No. 151, Guangwen Street, Kuiwen District, Weifang, 261000 Shandong People’s Republic of China

**Keywords:** Lung adenocarcinoma, LINC00963, HNRNPA2B1, Siah1, Zeb1, Exosomes, M2 macrophage polarization

## Abstract

**Background:**

Long intergenic non-coding RNA 00963 (LINC00963) is an oncogenic lncRNA in human cancers. However, little is known on how it impacts the pathogenesis of lung adenocarcinoma (LUAD).

**Methods:**

Biological effects on proliferation, migration, invasion, and epithelial-mesenchymal transition (EMT) were examined by CCK-8, colony formation, EdU incorporation, transwell, and immunofluorescence assays, respectively. Macrophage polarization was evaluated by flow cytometry. Ubiquitination of Zeb1 was examined by co-immunoprecipitation. The location of LINC00963 in LUAD tissues and cell lines was tested by FISH assay. The LINC00963/HNRNPA2B1/Siah1 mRNA complex interaction was verified using RNA pull-down and immunoprecipitation assays. The exact roles of LINC00963 were further validated in metastasis and xenograft models.

**Results:**

Higher LINC00963 expression in LUAD patients positively correlated with shorter overall survival, higher stages, and metastasis. LINC00963 mainly localized in the cytoplasm and aggravated malignant phenotypes of LUAD cells in vitro and metastasis in vivo. Mechanistically, LINC00963 directly interacted HNRNPA2B1 protein to trigger the degradation of Siah1 mRNA, which inhibited the ubiquitination and degradation of Zeb1. Moreover, exosomal LINC00963 derived from LUAD cells induced M2 macrophage polarization and promoted LUAD growth and metastasis.

**Conclusion:**

By stabilizing Zeb1 in cancer cells and delivering exosomes to induce M2 macrophage polarization, LINC00963 promoted the malignancy and metastasis of LUAD. Targeting LINC00963 may become a valuable therapeutic target for LUAD.

## Introduction

Despite significant advances in its mechanistic understanding, diagnosis, and treatment, the morbidity and mortality of lung cancer remain No. 1 among all cancers and are still on the rise (Bade and Dela Cruz 2020). These trends are reflected on the parallel increase in lung adenocarcinoma (LUAD), the most common histological subtype of lung cancer (Barta et al. 2019), implying the importance of continuous efforts to reveal pathogenic mechanisms of LUAD and to develop corresponding strategies. Cancer progression is driven collectively by malignant behaviors intrinsic to tumor cells, such as proliferation, migration, and invasion, as well as their extrinsic interactions with components from the tumor microenvironment. For example, there is close crosstalk between tumor cells and tumor-associated macrophages, an abundant population of immune cells in lung cancer characterized by immunosuppressive M2 phenotypes and dictating worse prognosis of cancers (Ge and Ding 2020). Therefore, identifying mechanisms simultaneously regulating malignant behaviors and inducing M2 polarization of macrophages will provide ideal targets for therapeutic intervention in LUAD.

Cumulative evidence suggests that long non-coding RNAs (lncRNAs) are not useless transcriptional products, but instead master regulators of many cancer hallmarks (Davalos and Esteller 2019; Schmitt and Chang 2016). Mechanistically, lncRNAs may act on chromatin, transcriptional, post-transcriptional, and post-translational levels, which are primarily determined by their localization in different subcellular compartments, including nucleus, cytoplasm, and exosomes (Carlevaro-Fita and Johnson 2019). Exosomes are extracellular vesicles of average 100 nm in diameter that serve as vehicles for delivering RNAs, DNAs, and proteins between cells and play crucial roles in every step of tumor development (Dai et al. 2020). The research on cancer cells-derived exosomal lncRNAs is still in its infancy, although earlier findings support their significance as cancer biomarkers, prognosis indicators, or active players in regulating malignant phenotypes (Dragomir et al. 2018).

The focus of this study is the long intergenic non-coding RNA 00963 (LINC00963), a lncRNA presenting oncogenic activities in a variety of human cancers. By far, studies on LINC00963 in cancers have revealed its mechanism by functioning as a competing endogenous RNA (ceRNA), that is, through sponging microRNAs (miRNAs) (Jiao et al. 2018; Lee et al. 2020; Liu et al. 2020a, b; Ye et al. 2020; Zhang et al. 2019; Zheng and Zhang 2020; Zhou et al. 2020). The only non-ceRNA mechanism identified for LINC00963 was from a study, where one group reported that by activating AKT/mTOR pathway and CREB-mediated transcription, LINC00963 drived the malignant progression of non-small cell lung cancer (Yu et al. 2017). Here, we examined the clinical significance of LINC00963 and its biological effects on the malignancy of LUAD, and investigated the impacts of exosomal LINC00963 on the polarization of macrophages. This study corroborates the oncogenic nature of LINC00963 in LUAD and reveals two novel mechanisms responsible for its pro-tumor activities: stabilizing Zeb1 to promote epithelial-mesenchymal transition (EMT) and delivering exosomes to induce M2 macrophage polarization.

## Materials and methods

### Collection of clinical samples

Sixty matching pairs of LUAD tissues and noncancerous lung tissues were obtained from patients during surgery. All diagnoses were confirmed by pathological examination. Written consents were signed by all patients and this study was approved by the Ethics Committee of Affiliated Hospital of Jining Medical University. The clinicopathological features of all patients are summarized in Table 1.

**Table 1:** The clinicopathological features of lung adenocarcinoma patients

Clinicopathological features	Cases (n)
Age (years)	
> 60	31
≤ 60	29
Gender	
Female	12
Male	48
TNM stage	
I–II	19
III–IV	41
Lymph node metastasis	
Yes	34
No	26
Smoking status	
Yes	42
No	18
Tumor size (cm)	
> 5	33
≤ 5	27

### Cell culture

The human non-tumorigenic lung epithelial cell line BEAS-2B and LUAD cell lines, NCI-H1975, NCI-H1299, A549, Calu-3, NCI-H358, and NCI-H1650 were ordered from the Cell Bank of Type Culture Collection of Chinese Academy of Sciences (Shanghai, China). All cells were cultured in α-MEM medium supplemented with 10% fetal bovine serum and 1% penicillin/streptomycin (Invitrogen, Carlsbad, CA, USA).

Peripheral blood mononuclear cells (PBMCs) were isolated from healthy donors using Ficoll density gradient centrifugation. Isolation of monocytes from human PBMCs was performed using human Pan Monocyte Isolation Kit (Miltenyi Biotec, Bergisch Gladbach, Germany). To generate monocyte-derived macrophages (MDMs), recombinant human macrophage colony stimulating factor (M-CSF; 30 ng/mL; Sigma-Aldrich, St. Louis, MO, USA) was added to the culture medium of monocytes at 37 °C for 7 days. To induce M2 macrophage polarization, fresh RPMI-1640 (Invitrogen) containing IL-4 and IL-13 (20 ng/mL; Sigma-Aldrich) was added to cells for a further 48 h.

### Fluorescence in-situ hybridization (FISH)

The localization of LINC00963 in LUAD tissues and cell lines were detected using FISH Tag RNA Multicolor Kit (Thermo Fisher Scientific, Waltman, MA, USA) following the instruction and using a customized LINC00963 probe designed by GenePharma (Shanghai, China). The cells were then counterstained with DAPI (Thermo Fisher Scientific) and imaged under the confocal microscope (Carl Zeiss, Oberkochen, Germany).

### Establishing stable cells and cell transfection

Lentivirus pLCDH vectors that overexpressing LINC00963 or distinct sh-LINC00963 (sh-LINC00963-1/2) were all generated by RiboBio (Guangzhou, China). Lentiviral vectors were transfected into 293T packing cells with other packaging vectors to generate lentiviral particles, which were then transduced into target A549 or NCI-H1650 at MOI of 10 with polybrene (10 µg/mL; Sigma-Aldrich). Cells were incubated for another 48 h and ready for further analyses.

To transiently knock down Zeb1 or overexpress Siah1 (OE-Siah1) in A549 or NCI-H1650 cells, sh-Zeb1 or pcDNA3.1-Siah1 (GenePharma) was transfected into LUAD cells using Lipofectamine 3000 (Invitrogen) in accordance with the manufacturer’s instructions. sh-NC and pcDNA3.1-NC (OE-NC) vectors were used as the corresponding negative controls.

### Cell proliferation assays

Short-term cell proliferation was examined using CCK-8 Kit (Sigma-Aldrich) and Cell-light EdU Apollo567 In Vitro Kit (RiboBio), respectively, following the manufacturer’s protocols.

For long-term cell proliferation, 200 LUAD cells were seeded into 24-well plate and cultured for 10 days. Cell colonies were stained with 0.2% crystal violet and counted under a light microscope (Carl Zeiss).

### Cell migration and invasion assays

Cell migration and invasion were examined using non-coated (for migration) or pre-coated Transwell inserts (Corning, Lowell, MA, USA) as detailed previously (Justus et al. 2014). Migrated or invaded cells were stained with 0.2% crystal violet, photographed (Carl Zeiss), analyzed using NIH Image J software.

### Immunofluorescence of E-cadherin and N-cadherin

Cells on glass coverslips were fixed in 4% paraformaldehyde and permeabilized with ice-cold 100% methanol. After incubating cells with primary mouse anti-E-cadherin (ab76055) and rabbit anti-N-cadherin (ab76011) antibodies (1:1000; both from Abcam, Cambridge, MA, USA) at 4 °C overnight, signals were detected with Alexa Fluor 488 conjugated anti-mouse and Alexa Fluor 647 conjugated anti-rabbit secondary antibodies (Abcam). Cells were then mounted with Vectashield Antifade Mounting Medium with DAPI (Vector Labs, Burlingame, CA, USA) and imaged under a fluorescence microscope (Carl Zeiss).

### Cycloheximide (CHX) chase assay

The stability of Zeb1 protein was measured by cycloheximide chase assay as detailed in a previous study (Kao et al. 2015). In short, A549 cells were incubated with complete medium containing cycloheximide (0.1 mg/mL) for indicated time periods (up to 8 h). Zeb1 protein level was then measure by western blot.

### RNA immunoprecipitation (RIP) assay

RIP assay was performed using Imprint RNA Immunoprecipitation Kit (Sigma-Aldrich) according to the manufacturer’s instruction. Briefly, LUAD cell lysates were incubated with anti-Zeb1 antibody (ab276129, Abcam), anti-Siah1 (MA5-24781, Invitrogen), anti-HNRNPA2B1 (ab31645, Abcam), or the non-specific anti-IgG antibody (Abcam) at 4 °C overnight. Protein-RNA complexes were then captured using protein A magnetic bead and associated RNAs were analyzed by RT-qPCR.

### RNA pull-down assay

RNA pull-down assay was performed using biotin-labeled full-length LINC00963 and its antisense RNA transcript as described previously (Zhang et al. 2017). In short, after incubating whole cell lysates from target LUAD cells with biotin-labeled RNA probes, RNA-associated proteins were captured with streptavidin Sepharose bead (GE Healthcare) and then analyzed by western blot.

### Measurement of mRNA stability

Upon pulsing A549 or NCI-H1650 cells with Actinomycin D (2.5 µg/mL) for different time periods, mRNA stability of Siah1 was measured by RT-qPCR and normalized to the level of GAPDH.

### Exosome isolation and labeling

Medium from A549 cells was first filtered through a PVDF filter (0.22 μm; Millipore, MA, USA) and then used for exosome isolation following the protocol of ExoQuick-TC Kit (System Biosciences). PKH67 Green Fluorescent Cell Linker Kit for General Cell Membrane Labeling (Sigma-Aldrich) was used to label the isolated exosomes.

### Transmission electron microscopy (TEM) and nanoparticle tracking analyzing (NTA)

Isolated exosomes were examined under a Zeiss EM109 TEM (Carl Zeiss) as detailed previously (Wu et al. 2015). The size distribution of isolated exosomes was measured using a Nano Sight NS500 instrument (Malvern Instruments Ltd, Malvern, UK) (Ruf et al. 1989).

### Flow cytometry

To measure the uptake of exosomes by macrophages, we incubated PKH67-labelled exosomes (40 µg/mL) with macrophages at 37 °C for 24 h. As the negative control, non-labeled exosomes were used. To analyze the polarization of macrophages, after incubating macrophages with exosomes (40 µg/mL) isolated from indicated A549 cells at 37 °C for 24 h, macrophages were stained with PE-labeled anti-CD206, anti-HLA-DR, or the corresponding isotype control IgG (all from BioLegend, San Diego, CA, USA). Flow cytometry was performed on FACSCalibur (BD Biosicences, New Jersey, USA).

### Reverse transcription followed by quantitative real-time PCR (RT-qPCR)

After extracting total RNA using Trizol reagent (Invitrogen) and synthesizing cDNA with PrimeScript RT Reagent Kit (Takara, Dalian, China), respectively. qPCR analysis was performed using iQTM SYBR Green Supermix (Bio-Rad, Hercules, CA, USA). The relative expression levels of target genes were normalized to the internal controls GAPDH or U6 and calculated using the 2^− ΔΔCt^ method (Livak and Schmittgen 2001). The relative expression of LINC00963 in LUAD tissues was normalized to the average expression of the matching para-tumor normal tissues. Primers for RT-qPCR were purchased from Sangon Biotech (Shanghai, China).

### Cell fractionation, co-immunoprecipitation (Co-IP), and western blot

Cell fractionation was performed using Cell Fractionation Kit (Abcam) following the manufacturer’s instruction. To detect ubiquitination of Zeb1, whole cell lysates were prepared in IP lysis buffer (Thermo Fisher Scientific), incubated with anti-Zeb1 antibody (ab276129, Abcam) or rabbit IgG on a rotating shaker at 4 °C for 1 h, followed by protein A Sepharose bead (Abcam) at 4 °C overnight. After washing the beads and associated protein complexes for three times, they were boiled in sample loading buffer for 5 min and the supernatant was examined using western blot.

For western blot, RIPA buffer was used to extract total proteins. Protein concentration was quantified by BCA Protein Assay Kit (Thermo Fisher Scientific). After electrophoresis through SDS-PAGE gel, proteins were transferred to PVDF membranes, blocked in TBST with 5% non-fat milk at room temperature for 1 h, and blotted with primary antibodies for E-cadherin (1:1000; ab40772), N-cadherin (1:2000; ab76011), Vimentin (1:1500; ab92547), Zeb1 (1:1000; ab276129), Snail (1:3000; ab216347), Slug (1:3000; ab106077), Twist (1:1000; ab50887), Ubiquitin (1:1000; ab134953), HNRNPA2B1 (1:2000; ab31645), Siah1 (1:2000; ab2237), CD63 (1:3000; ab134045), TSG101 (1:1500; ab125011), CD9 (1:1500; ab236630), GM130 (1:2000; ab52649), Calnexin (1:1000; ab133615), Lamin B1 (1:1500; ab16048; internal control), or GAPDH (1:5000; ab8245; internal control) at 4 °C overnight. Upon incubating with horseradish peroxidase-conjugated anti-mouse (1:5000; ab6728) or anti-rabbit (1:5000; ab6721) secondary antibodies, target signals were developed with ECL substrate (Beyotime, Jiangsu, China).

### Animal experiments

Procedures on experimental animals were pre-approved by the Institutional Animal Care and Use Committee of Affiliated Hospital of Jining Medical University. Six-week-old male BALB/C nude mice were purchased from SLAC Laboratory Animal Center (Shanghai, China) and maintained in a standard facility. To monitor tumor metastasis in vivo, indicated A549 cells (OE-NC, OE-LINC00963, sh-NC, or sh-LINC00963) were injected into the tail vein (1 × 10^6^ cells/mouse in 100 µL of saline, n = 10 per group). The mice were sacrificed with lungs isolated, measured for the wet weight, and imaged before being fixed in 10% formalin. The macroscopic metastatic nodules were counted.

To establish xenograft model, 5 × 10^5^ MDMs pre-treated with indicated exosomes were subcutaneously injected together with non-transfected 5 × 10^5^ A549 cells (n = 6 per group). Every 3 days thereafter, tumor length (L) and width (W) were measured with a caliper and tumor volume (V) was calculated as: V = L×W^2^ × 1/2. On day 21, all mice were euthanized and xenografts were isolated for further analyses.

### Histological analyses

Lung or tumor tissues from mice were fixed in 10% formalin and embedded in paraffin. Hematoxylin and eosin (HE) staining was performed using Hematoxylin and Eosin Stain Kit (Vector Labs) following the manufacturer’s protocol. For immunohistochemical staining of Ki-67, Zeb1, and CD206, tumor tissue sections were boiled with 10 mM citrate buffer (pH 6.0) for 10 min to retrieve antigen. After blocking tissues by 5% normal goat serum, anti-Ki-67 (1:500; ab15580), anti-Zeb1 (1:200; ab203829), or anti-CD206 (1:200; ab64693, all from Abcam) antibodies were applied, followed by biotin-labeled secondary antibody and ABC-HRP solution (Vector Labs). Target signals were developed using Diaminobenzidine (DAB) substrate (Vector Labs).

### Statistical analysis

All experiments were independently repeated at least three times and data were presented as mean ± standard deviation (SD). Statistical analysis was accomplished using GraphPad Prism 6.0. All the data meet the assumption of Gaussian distribution. Differences between two groups was examined using Student’s *t*-test and those from more than two groups using one-way analysis of variance (ANOVA) followed by Tukey post hoc test. The correlation between LINC00963 and HNRNPA2B1 expression levels was analyzed using Pearson correlation assay. The association between LINC00963 level and overall survival was examined by Kaplan-Meier Survival method. Significance was defined as *P* < 0.05.

## Results

### LINC00963 was up-regulated and positively associated with worse prognosis of LUAD patients

**Fig. 1:**
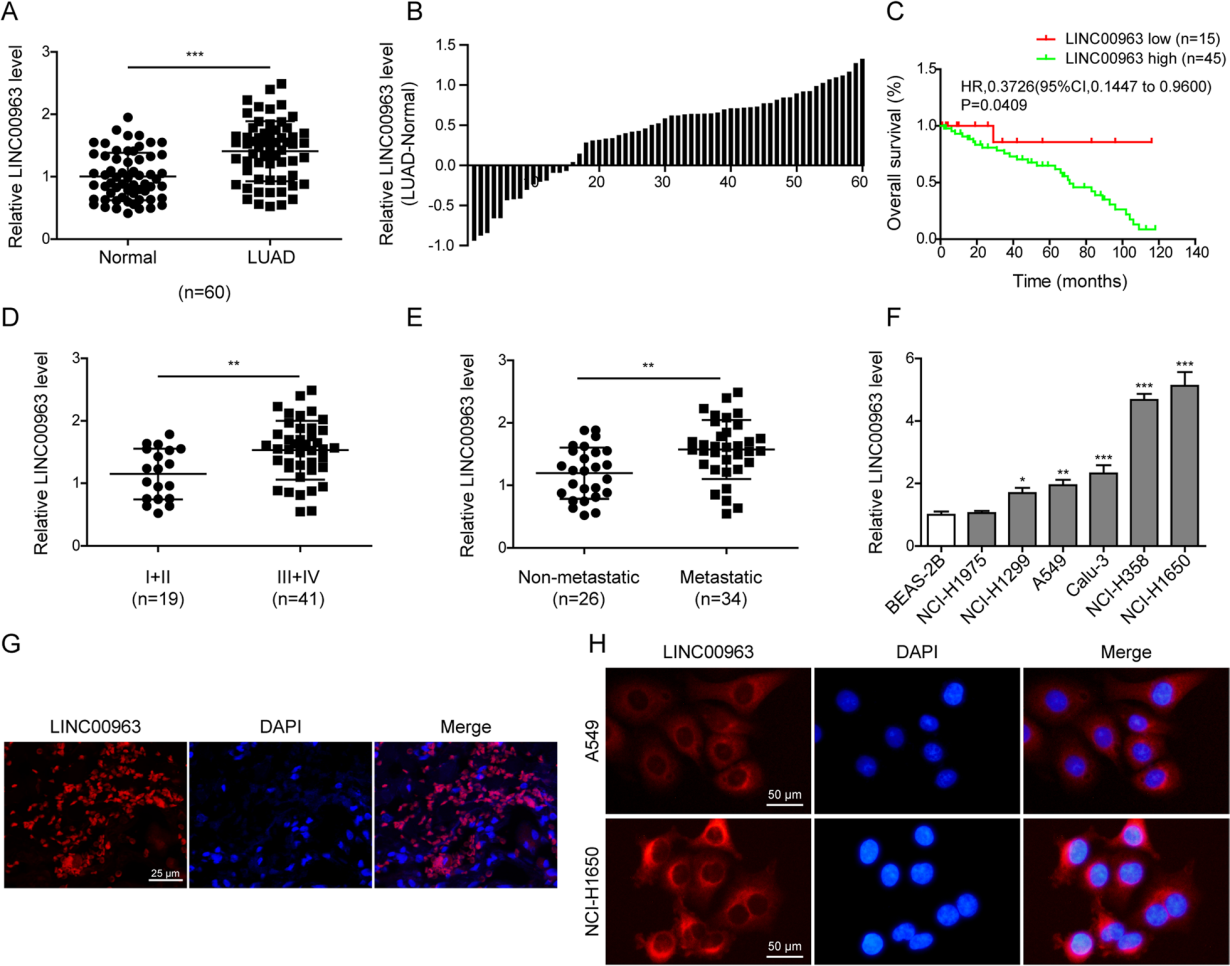
LINC00963 was up-regulated and positively associated with worse prognosis of LUAD patients. **A** LINC00963 expression levels were examined by RT-qPCR between 60 pairs of LUAD tissues and matching noncancerous lung tissues. **B** The expression level of LINC00963 tested in A was presented with each individual patient. **C** The overall survival was analyzed between LUAD patients presenting high (n = 45) and low (n = 15) LINC00963 expression using Kaplan-Meier method. **D** LINC00963 expression was compared between LUAD tissues of higher (III + IV; n = 41) and lower (I + II; n = 19) TNM stages. **E** LINC00963 expression was compared between non-metastatic (n = 26) and metastatic (n = 34) LUAD tumors. **F** LINC00963 expression was examined by RT-qPCR in lung epithelial cell line BEAS-2B and six LUAD cell lines, NCI-H1975, NCI-H1299, A549, Calu-3, NCI-H358 and NCI-H1650. **G**, **H** FISH analysis of LINC00963 in LUAD tissues (**G**), and in A549 and NCI-H1650 cells (**H**). *P < 0.05, **P < 0.01 and ***P < 0.001

To understand the clinical importance of LINC00963 in LUAD, we first compared its expression between 60 matching pairs of LUAD and normal pulmonary tissues. The average LINC00963 expression level was significantly higher in LUAD tissues (Fig. 1A), and the LINC00963 expression level was up-regulated (as indicated by the LUAD-normal expression > 0) in 45 cases, and down-regulated in 15 cases (Fig. 1B). Analysis of clinicopathological features of LUAD patients demonstrated that LINC00963 levels were positively correlated with shorter overall survival (Fig. 1C), and were significantly higher in advanced tumors (TNM stage: III + IV, n = 41, Fig. 1D) and metastatic tumors (n = 34, Fig. 1E). These data revealed the significant association between higher LINC00963 expression level and the malignant progression of LUAD in clinic. Further expressional analysis in several cancer cell lines showed that when compared to the non-tumorigenic lung epithelial cell line BEAS-2B, LINC00963 was moderately up-regulated in NCI-H1299, A549, Calu-3 cells and robustly up-regulated in NCI-H358 and NCI-H1650 cells (Fig. 1F). To further investigate the biological functions and molecular mechanisms of LINC00963 in LUAD, we chose A549 and NCI-H1650 cells as the in vitro model for further analyses. To map the location of LINC00963, we applied FISH and detected cytoplasmic expression of LINC00396 both in LUAD tissues (Fig. 1G) as well as in lung cancer cells (Fig. 1H).

### LINC00963 sufficiently induced the proliferation, migration, invasion, and EMT of LUAD cells

**Fig. 2:**
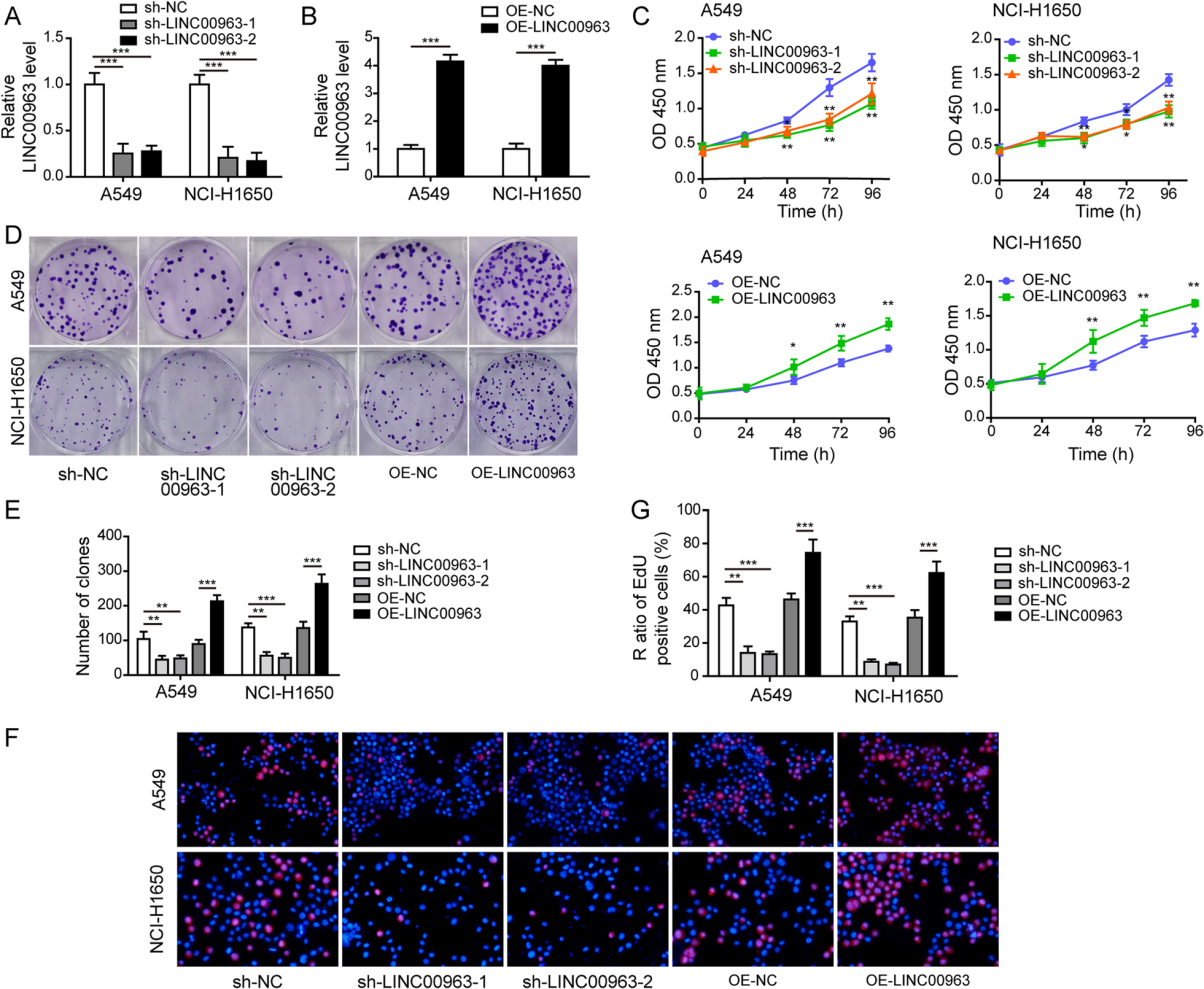
LINC00963 sufficiently induced the proliferation of LUAD cells. A549 and NCI-H1650 cells stably expressed two distinct shRNA targeting LINC00963 or overexpressed LINC00963. **A**, **B** RT-qPCR assay examined LINC00963 expression in indicated cells with LINC00963 overexpression or silence. Proliferation (**C**), long-term proliferation (**D**, **E**), and short-term proliferation (**F**, **G**) of indicated cells were determined using CCK-8, colony formation, and EdU assays, respectively. *P < 0.05, **P < 0.01 and ***P < 0.001

**Fig. 3:**
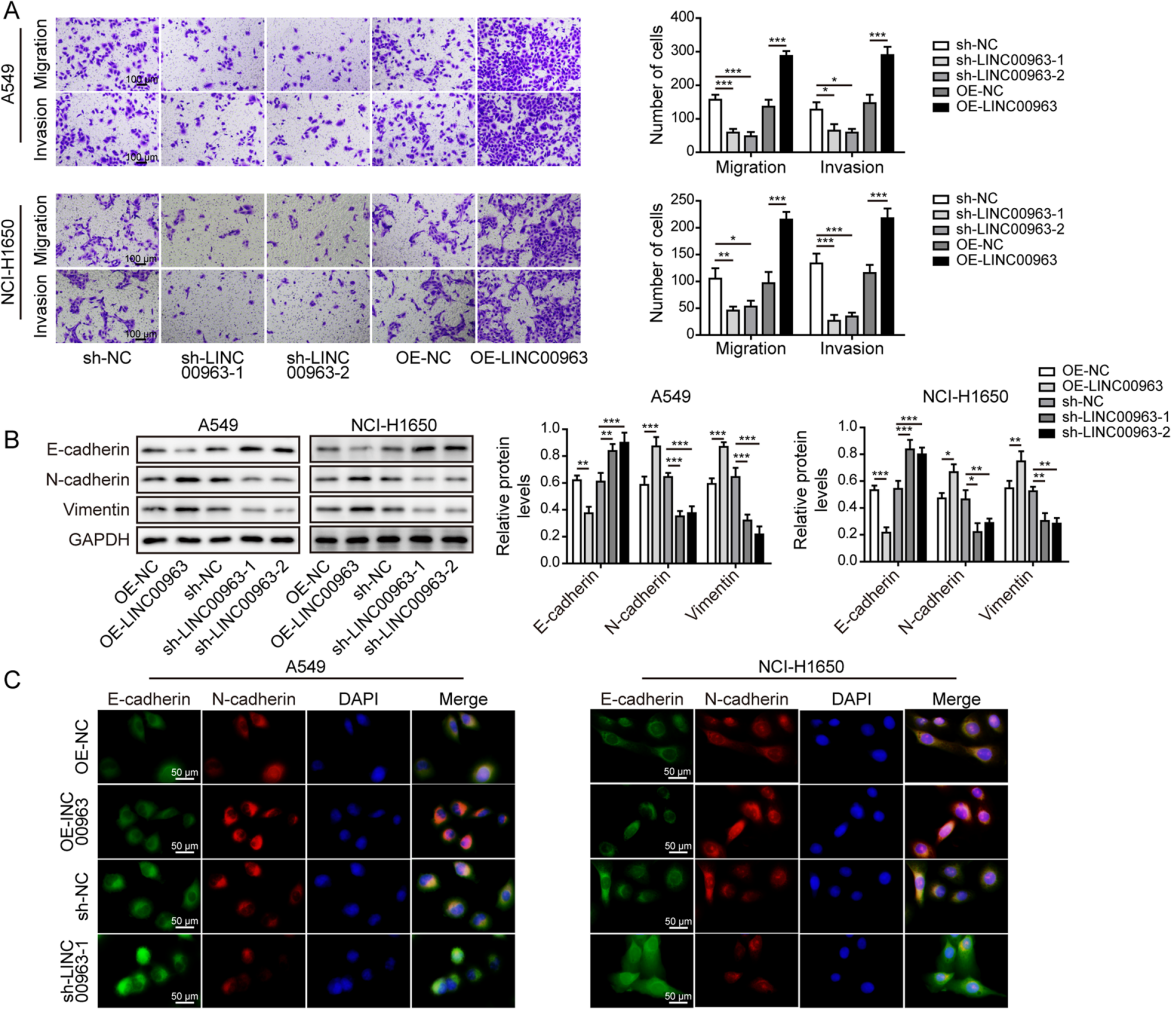
LINC00963 significantly induced the migration, invasion, and EMT of LUAD cells. A549 and NCI-H1650 cells stably expressed two distinct shRNA targeting LINC00963 or overexpressed LINC00963. **A** Cell migration and invasion were assessed by Transwell assays. **B** Expression levels of E-cadherin, N-cadherin, and vimentin were examined by western blot. **C** E-cadherin (green) and N-cadherin (red) were detected by immunofluorescence and the nuclei were stained by DAPI (blue) in indicated cells. Scale bar: 50 μm. *P < 0.05, **P < 0.01 and ***P < 0.001

To assess the biological functions of LINC00963 in LUAD cells, we stably knocked down or overexpressed LINC00963 expression in A549 and NCI-H1650 cells. Both sh-LINC00963-1 and sh-LINC00963-2 transfection drastically reduced the expression of LINC00963 (Fig. 2A), and LINC00963 overexpressing cells demonstrated approximately 4-fold expression of LINC00963 (Fig. 2B). The cell proliferation (Fig. 2C–G), migration and invasion (Fig. 3A), and the expression levels of N-cadherin and vimentin (Fig. 3B, C) were all significantly reduced in sh-LINC00963-1/2 cells, but markedly higher in LINC00963 overexpressing cells, excepted the expression level of E-cadherin (Fig. 3B, C). These results suggested that LINC00963 played an essential and sufficient role in promoting multiple malignant behaviors of LUAD cells in vitro.

### LINC00963 crucially enhanced tumor metastasis in vivo

**Fig. 4:**
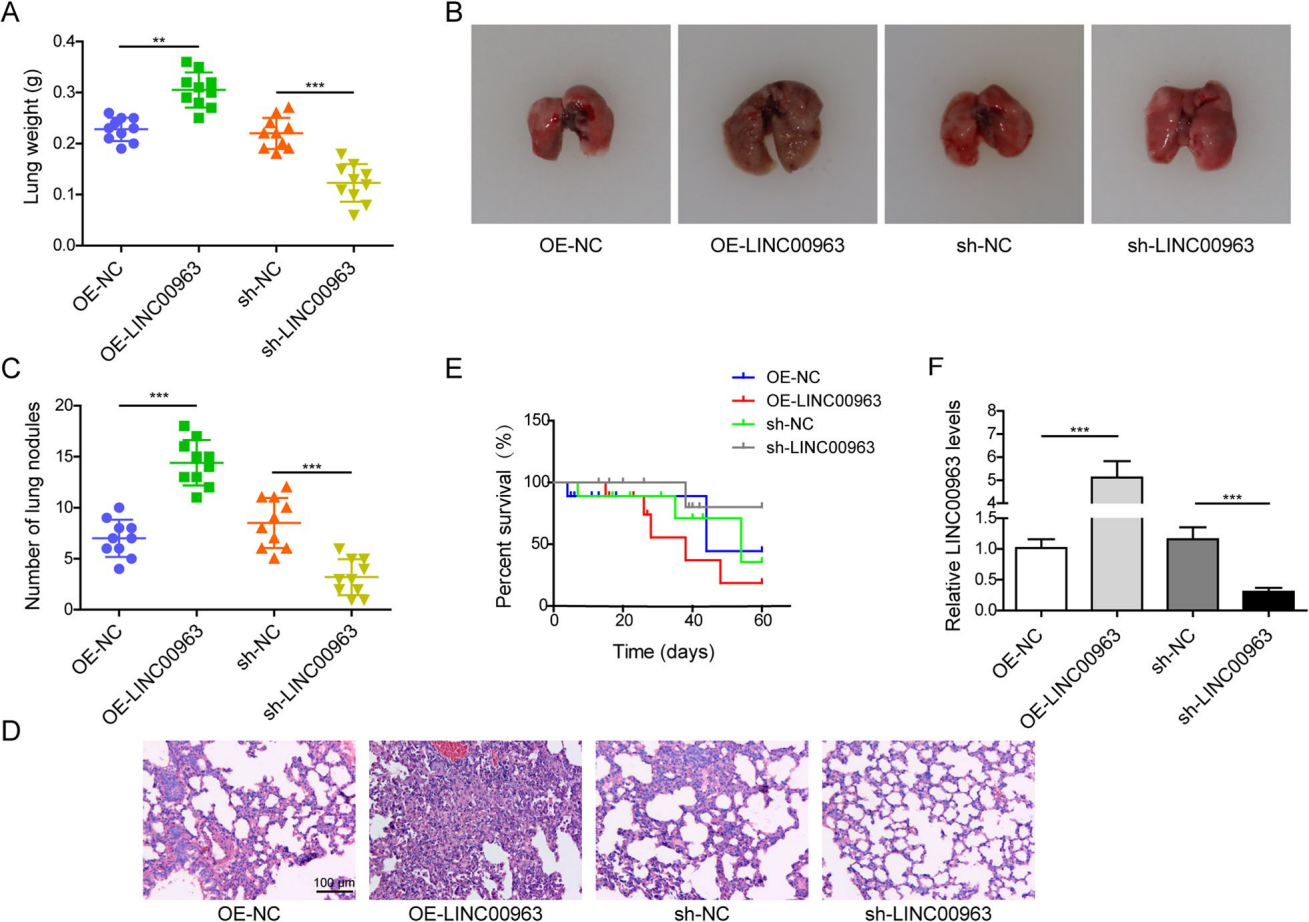
LINC00963 crucially enhanced tumor metastasis in vivo. A549 cells with LINC00963 overexpression or silence were injected intravenously into nude mice. **A** The weights of lung tissues were measured between indicated groups of mice. **B** Photographs were taken on lung tissues from indicated groups. **C** The number of lung metastatic nodules was analyzed between indicated groups of mice. **D** The HE staining was performed on lung tissues from indicated groups. Scale bar: 100 μm. **E** The overall survival was compared between indicated groups using Kaplan–Meier method. **F** LINC00963 level was measured by RT-qPCR in lung tissues from indicated groups. **P < 0.01 and ***P < 0.001

The in vitro phenotypes examined above supported that LINC00963 promoted the growth and mobility of LUAD cells. To understand whether LINC00963 critically regulates tumor metastasis in vivo, we intravenously injected A549 cells with LINC00963 overexpression or silence vector into immunocompromised nude mice. The weights of lungs were significantly higher with LINC00963 overexpression, and were the lowest in those with LINC00963 silence (Fig. 4A). Correspondingly, the highest number of macroscopic metastatic nodules was detected in lungs with LINC00963 overexpression, and the lowest in those with LINC00963 knock down (Fig. 4B, C). Histological analysis showed the similar pattern of microscopic metastatic nodules in lungs (Fig. 4D). The difference in metastatic nodules also affected the overall survival of mice, with the shortest survival observed in mice with LINC00963 overexpression and the longest in those with LINC00963 silence (Fig. 4E). Expressional analysis validated significant up-regulation or down-regulation of LINC00963 expression in lungs from LINC00963 overexpression or silence group (Fig. 4 F). Therefore, these data suggested that LINC0000963 not only sufficiently but also necessarily drived the in vivo metastasis and shortened the survival of mice in LUAD.

### LINC00963 stabilized Zeb1 by modulating its ubiquitination

**Fig. 5:**
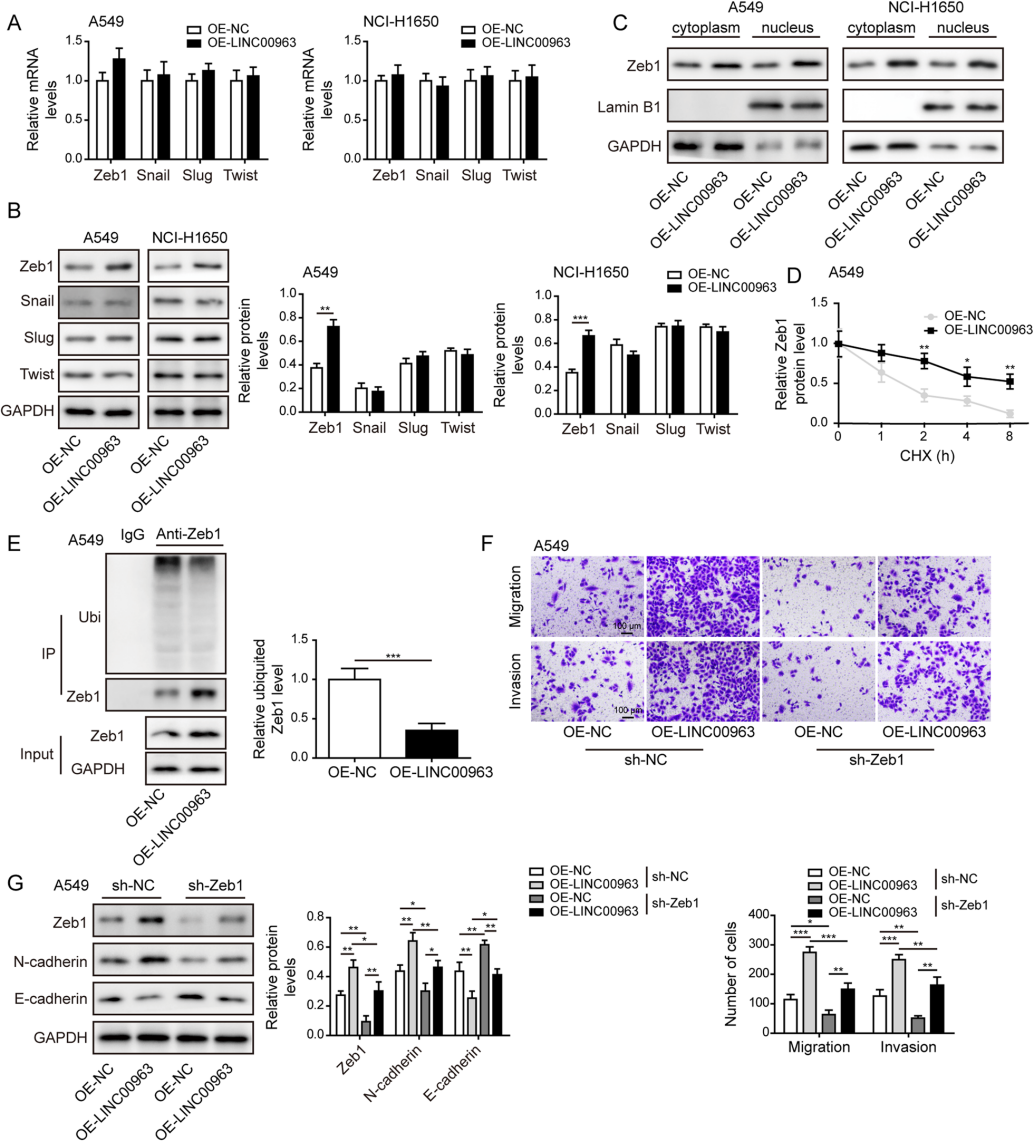
LINC00963 stabilized Zeb1 by modulating its ubiquitination. Expression levels of Zeb1, Snail, Slug, and Twist in LINC00963 overexpressed cells were examined by RT-qPCR (**A**) and western blot (**B**). **C** Cytoplasmic and nuclear fractions were prepared from A549 and NCI-H1650 cells. Zeb1 expression was examined by western blot. **D** Stability of Zeb1 protein was examined by CHX chase assay in LINC00963 overexpressed A549 cells. **E** Ubiquitinated Zeb1 was measured by Co-IP assay in LINC00963 overexpressed A549 cells. **F** Migration and invasion were assessed by Transwell assays in indicated A549 cells. Scale bar: 100 μm. **G** Western blot was performed to test Zeb1, E-cadherin, and N-cadherin protein levels. *P < 0.05, **P < 0.01 and ***P < 0.001

To explore mechanisms underlying the oncogenic activities of LINC00963, we focused on EMT, the biological process critical for cancer metastasis. We detected no appreciable differences in the mRNA levels of several core EMT-related transcriptional factors, including Zeb1, Snail, Slug, and Twist with LINC00963 overexpression (Fig. 5A). However, LINC00963 overexpression significantly up-regulated Zeb1 protein level, but not Snail, Slug, or Twist protein level (Fig. 5B). Ectopic expression of LINC00963 also ubiquitously elevated Zeb1 protein level in both the cytoplasm and nucleus (Fig. 5C). Stability analysis revealed that LINC00963 overexpression significantly increased the half-life of Zeb1 protein under CHX treatment (Fig. 5D). Since ubiquitination regulates the stability of Zeb1 protein (Chen et al. 2015; Xu et al. 2015), we examined Zeb1 ubiquitination level in LINC00963 overexpressed A549 cells. LINC00963 overexpression significantly reduced the ubiquitinated level of Zeb1 protein (Fig. 5E). Transwell assays showed LINC00963 significantly promoted migration and invasion of A549 cells, which was partly abolished by sh-Zeb1 (Fig. 5F). Expressional analysis showed that sh-Zeb1 potently reversed the protein levels of Zeb1, N-cadherin, and E-cadherin caused by LINC00963 overexpression (Fig. 5G). The above results suggested that LINC00963 stabilized Zeb1 by targeting its ubiquitination, which mediated the effects of LINC00963 on migration, invasion and EMT.

### LINC00963 triggered the degradation of Siah1 mRNA to induce up-regulation of Zeb1

**Fig. 6:**
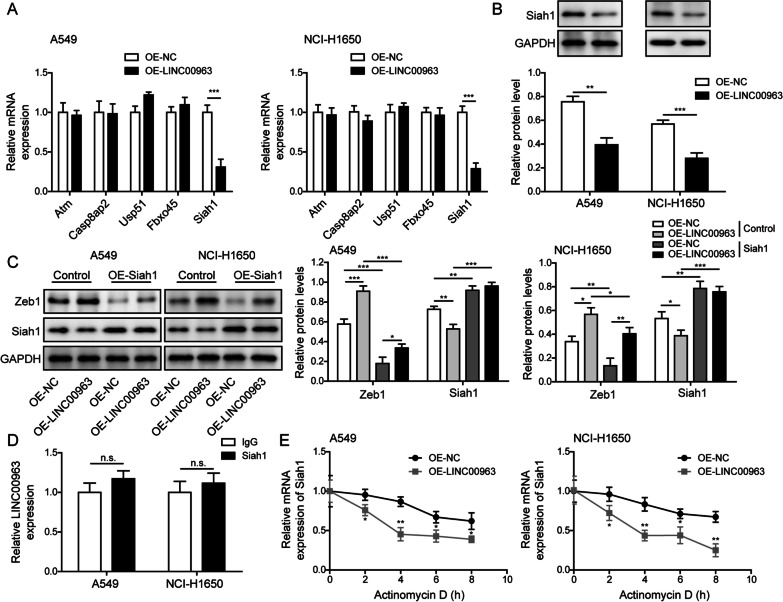
LINC00963 triggered the degradation of Siah1 mRNA to induce up-regulation of Zeb1. **A** Expression levels of indicated E3 ubiquitin ligases were examined by RT-qPCR in LINC00963 overexpressed cells. **B** Siah1 protein level was examined by western blot in LINC00963 overexpressed cells. **C** Expression levels of Zeb1 and Siah1 were examined by western blot in indicated cells. **D** The potential interaction between LINC00963 and Siah1 was examined by RIP assay using anti-Siah1 and IgG (negative control) antibody. **E** The mRNA stability of Siah1 was examined by RT-qPCR after treating Actinomycin D for indicated time periods in LINC00963 overexpressed cells. *n.s.*, not significant, *P < 0.05, **P < 0.01 and ***P < 0.001

Emerging evidence suggests that lncRNA-protein complex regulates gene expression on multiple levels (Rinn and Chang 2012). To understand whether LINC00963 alters the protein stability of Zeb1, we focused on a panel of E3 ubiquitin ligases reported to regulate the ubiquitination of Zeb1: Atm, Casp8ap2, Usp51, Fbxo45, and Siah1. Only Siah1 mRNA was drastically down-regulated in LINC00963-overexpressing A549 or NCI-H1650 cells (Fig. 6A). The down-regulation of Siah1 by LINC00963 was also detected on the protein level (Fig. 6B). To test whether Siah1 mediates the degradation of Zeb1, we ectopically expressed Siah1 in LINC00963-overexpressing cells and indicated that Zeb1 was up-regulated, while Siah1 was down-regulated under LINC00963 overexpression. However, ectopic expression of Siah1 partly abolished the up-regulation of Zeb1 in these cells (Fig. 6C). RIP assay revealed no significant association between LINC00963 and Siah1 protein (Fig. 6D). When examining the mRNA stability of Siah1, we found that LINC00963 accelerated the degradation of Siah1 mRNA (Fig. 6E). Together, these data suggested that LINC00963 promoted the degradation of Siah1 mRNA and thus up-regulated Zeb1.

### LINC00963 physically interacted with HNRNPA2B1 protein

**Fig. 7:**
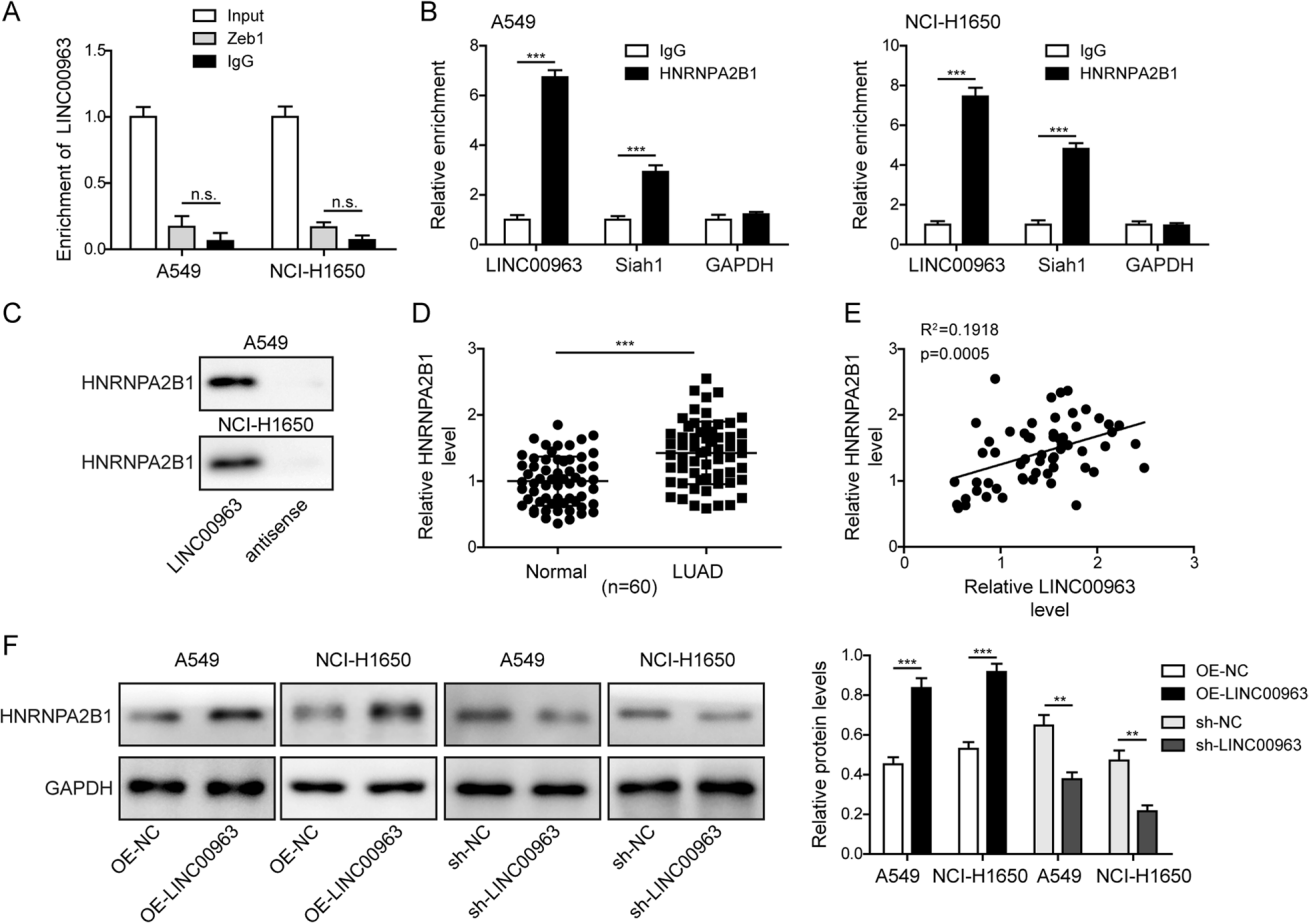
LINC00963 physically interacted with HNRNPA2B1 protein. **A** The potential interaction between LINC00963 and Zeb1 protein was assessed by RIP assay using anti-Zeb1 or IgG (negative control) antibody. **B** The potential interaction between HNRNPA2B1, LINC00963, and Siah1 mRNA was examined by RIP assay using anti-HNRNPA2B1 and IgG (negative control) antibody. **C** RNA pull-down assay verified the interaction between LINC00963 and HNRNPA2B1 protein. **D** RT-qPCR measured HNRNPA2B1 expression between 60 pairs of LUAD tissues and matching noncancerous lung tissues. **E** The association between LINC00963 and HNRNPA2B1 levels in 60 LUAD tissues was analyzed by Pearson correlation analysis. **F** HNRNPA2B1 expression in indicated cells was examined by western blot. *n.s.*, not significant, *P < 0.05, **P < 0.01 and ***P < 0.001

To further explore the mechanism mediating LINC00963-induced degradation of Siah1 mRNA and Zeb1 ubiquitination, we first measured the potential interaction between LINC00963 and Zeb1. RIP assay showed that LINC00963 did not directly interact with Zeb1 protein (Fig. 7A). Bioinformatic analysis using Starbase and RIP assay respectively predicted and revealed that both LINC00963 and Siah1 mRNA specifically interacted with HNRNPA2B1 protein (Fig. 7B). We then performed the RNA pull-down assay using LINC00963 probe, and found LINC00963 interacted with HNRNPA2B1 protein (Fig. 7C). Similar to the expression status of LINC00963, HNRNPA2B1 was also higher in LUAD tissues than in noncancerous lung tissues (Fig. 7D), and its level positively correlated with that of LINC00963 (Fig. 7E). In both A549 and NCI-H1650 cells, LINC00963 overexpression sufficiently up-regulated, while LINC00963 silence reduced HNRNPA2B1 protein level (Fig. 7F). Collectively, these data suggested that LINC00963 directly interacted with HNRNPA2B1 and up-regulated its expression in LUAD cells and tissues.

### LUAD cells-derived exosomal LINC00963 induced M2 macrophage polarization

**Fig. 8:**
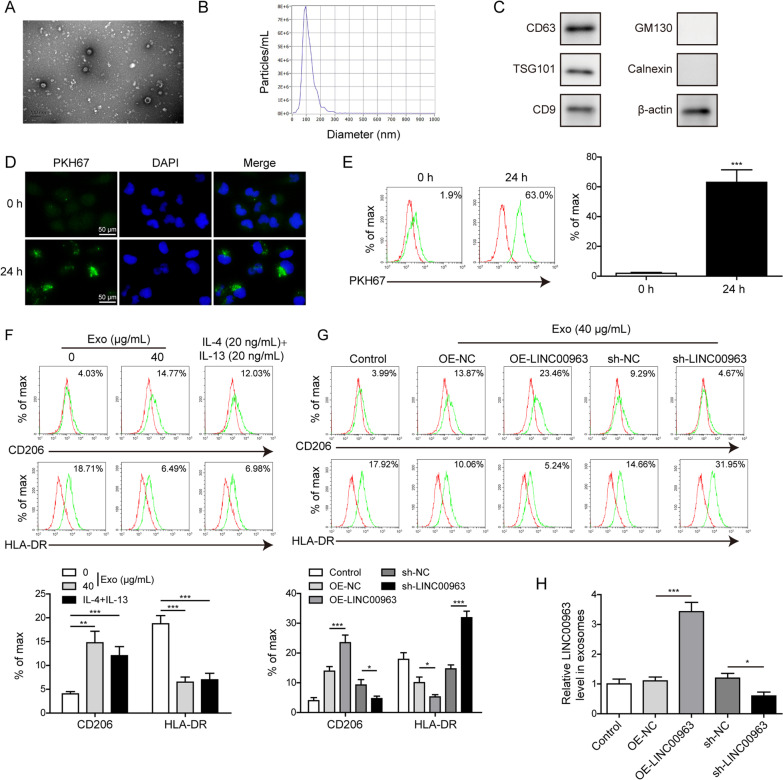
LUAD cells-derived exosomal LINC00963 induced M2 macrophage polarization.  Exosomes were isolated from the medium of A549 cells, observed for their morphology under TEM (**A**), measured for their size distribution by NTA (**B**), and analyzed for the expression levels of CD63, TSG101, CD9, GM130, calnexin by western blot (**C**). A549-derived exosomes were labeled with PKH67 and then incubated with macrophages. Macrophages were detected by immunofluorescence (**D**) or flow cytometry (**E**). Scale bar: 100 μm. **F** A549-derived exosomes 40 (µg/mL) were incubated with macrophages. Those incubated with IL-4 and IL-13 (20 ng/mL) were used as the positive control. Expression levels of CD206 and HLA-DR were examined by flow cytometry. **G** Exosomes isolated from indicated A549 cells were applied to macrophages. Expression levels of CD206 and HLA-DR on macrophages were examined by flow cytometry. **H** Exosomes were isolated from indicated A549 cells and examined for LINC00963 expression in exosomes by RT-qPCR. *P < 0.05, **P < 0.01, and ***P < 0.001

In addition to regulating gene expression, lncRNAs are also crucial contents in exosomes to help shaping tumor-promoting microenvironment (Sun et al. 2018). To understand whether LINC00963 is a cargo in LUAD cells-derived exosomes, we isolated exosomes from the conditioned medium. Under TEM, these exosome vesicles were approximately 100 nm in diameter (Fig. 8A), which were further confirmed by NTA (Fig. 8B). Expressional analysis showed that exosomal markers CD63, TSG101 and CD9, were all expressed on exosomes, while GM130 (a golgi apparatus marker) and calnexin (an endoplasmic reticulum marker) were not detected (Fig. 8C). To understand the functional consequence of exosomal LINC00963, we focused on macrophages, one of the most abundant immune cells within tumor microenvironment (Nielsen and Schmid 2017). When labeling exosomes with the green fluorescent dye PKH67, these exosomes were taken up by macrophages, as detected by immunofluorescence (Fig. 8D) and flow cytometry (Fig. 8E). Then we examined the impact of exosomes on macrophage polarization. Exosomes derived from LUAD cells increased the expression of M2 macrophage biomarker CD206 while decreased that of M1 biomarker HLA-DR to the similar extent as observed on macrophages treated with IL-4 and IL-13, inducers for M2 macrophages (Fig. 8F). Next, we isolated exosomes from cells with altered expression of LINC00963. As shown in Fig. 8G, exosomes from LINC00963 overexpression cells further boosted CD206 while reduced HLA-DR expression, while those from LINC00963 silence cells generated the opposite effects, suggesting that LUAD cells-derived exosomal LINC00963 critically stimulated M2 polarization of macrophages. Also, the LINC00963 expression was increased or decreased in exosomes after LINC00963 overexpression or knock down (Fig. 8H).

### Macrophages pre-conditioned with exosomal LINC00963 promoted LUAD growth in vivo

**Fig. 9:**
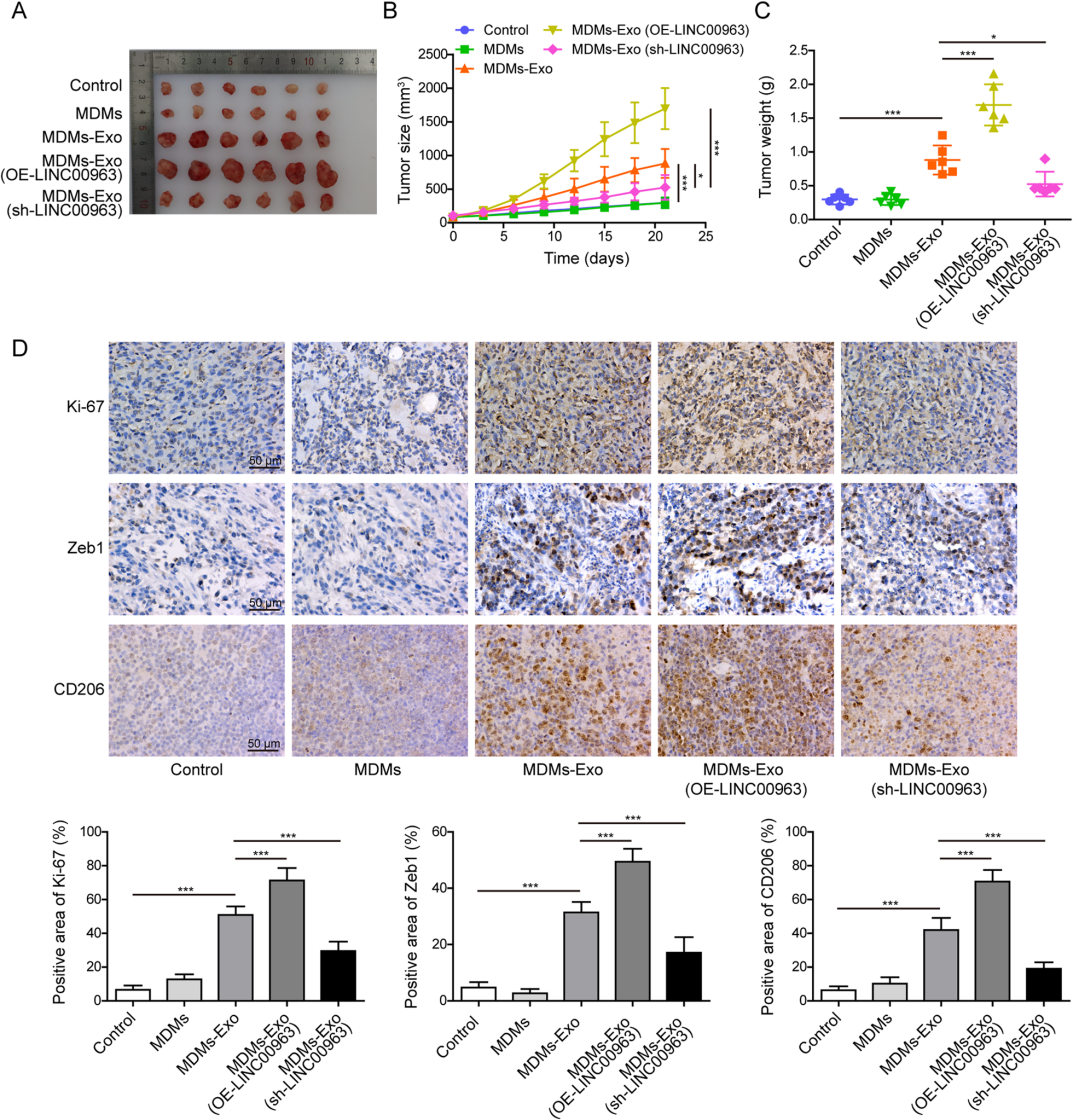
Macrophages pre-conditioned with exosomal LINC00963 promoted LUAD growth and metastasis in vivo. A549 cells, together with macrophages pre-treated with exosomes isolated from indicated A549 cells, were subcutaneously injected into nude mice. Photographs (**A**), growth curve (**B**), and weight (**C**) of xenografts from indicated groups. **D** Immunohistochemistry staining of Ki-67, Zeb1, and CD206 from indicated xenograft tumors. Scale bar: 50 μm. *P < 0.05 and ***P < 0.001

To assess in vivo impacts of macrophages pre-conditioned with exosomal LINC00963 on tumor progression in vivo, we firstly treated macrophages with exosomes isolated from A549 cells, or isolated from A549 cells with LINC00963 overexpression or knock down, and then injected A549 cells alone (control) or with exosomes-treated macrophages into immunocompromised mice. After 21 days, macrophages treated with normal exosomes or LINC00963 overexpression exosomes significantly induced the growth of xenografts, but LINC00963 silence exosomes exerted the inverse effects (Fig. 9A). The differences in the tumor size were also reflected on the growth curve (Fig. 9B) and the final weight (Fig. 9C) of xenografts. Immunohistochemical analysis revealed corresponding changes on Ki-67, Zeb1, and CD206 expression. The expression levels of all three proteins were reinforced by macrophages treated with normal exosomes or LINC00963 overexpression exosomes (Fig. 9D). Taken together, these data suggested that LUAD cells-derived exosomal LINC00963 promoted tumor growth in vivo via driving M2 macrophage polarization.

## Discussion

Metastasis is responsible for the majority of cancer-related death. However, the molecular mechanisms are still not well understood (Fares et al. 2020). Here we reported that LINC00963 not only was up-regulated in LUAD tissues, but correlated with LUAD patients of higher TNM stages, metastasis, and shorter overall survival. LINC00963 also critically promoted malignant and metastatic phenotypes of LUAD cells, both in vitro and *in vivo.* Furthermore, we revealed two novel mechanisms underlying the oncogenic activities of LINC00963. Firstly, through the interaction with HNRNPA2B1, LINC00963 aggravated the degradation of Siah1 mRNA, then stabilized Zeb1, and promoted EMT. Secondly, LUAD cells-derived exosomal LINC00963 induced M2 macrophage polarization and feedback to stimulate LUAD growth and metastasis.

LINC00963 is a lncRNA with well characterized oncogenic activities in a variety of human cancers, mostly in solid cancers (Jiao et al. 2018; Lee et al. 2020; Liu et al. 2020a, b; Ye et al. 2020; Zhang et al. 2019; Zheng and Zhang 2020; Zhou et al. 2019, 2020; Yu et al. 2017; Sun et al. 2020; Wang et al. 2014, 2019; Wu et al. 2018, 2020; Zhu et al. 2020), but also hematological cancers (Zuo et al. 2020). These studies also suggested the clinical association between higher LINC00963 level and poor prognosis of cancer patients, and its biological roles in promoting proliferation, migration, invasion, EMT, drug resistance, radio-resistance, and cancer stemness. Consistent with these findings, here we also reported the up-regulation of LINC00963 was associated with LUAD patients of advanced pathological stages, metastatic behavior, and shorter overall survival, demonstrating the clinical significance of LINC00963 in predicting poor prognosis of LUAD. Mechanistically, most studies by far have suggested LINC00963 acts through the lncRNA/miRNA/mRNA axis, such as miR-608/NACC1 in melanoma (Jiao et al. 2018), miR-214-5p/RAB14 in esophageal cancer (Liu et al. 2020a, b), miR-378 g/CHI3L1 in ovarian cancer (Liu et al. 2020a, b), miR-542-3p/NOP2 in prostate cancer (Sun et al. 2020), and miR-625/HMGA1 and miR-324-3p/ACK1 in breast cancer (Zhang et al. 2019; Wu et al. 2020). Although lncRNA/miRNA/mRNA axis is not the only mechanism by which lncRNAs function, the only study so far suggesting the mechanism of LINC00963 not dependent on miRNA/mRNA axis is from Yu et al. (2017), who showed that through physical interaction with PGK1, LINC00963 inhibited proteasome-induced degradation of the PGK1 and activated AKT/mTOR signaling. In addition, through the physical interaction with NONO, LINC00963 functioned as a co-activator for CRTC/CREB-regulated gene transcription (Yu et al. 2017). In this study, by using RIP and RNA pull-down assays, we identified HNRNPA2B1 as an interacting partner for LINC00963. Therefore, this is the first study showing the interaction between LINC00963 and HNRNPA2B1, the up-regulation of HNRNPA2B1 by LINC00963, and their positive correlations in LUAD tissues. Functionally, it is known that nuclear lncRNA-hnRNA interaction regulates genome structure, activates or represses gene transcription, while cytoplasmic lncRNA-hnRNA complex controls the stability or the translation of mRNAs (Sun et al. 2017). Consistently, here we showed LINC00963 mainly localized in the cytoplasm, and LINC00963/HNRNPA2B1 complex promoted the degradation of Siah1 mRNA and consequently up-regulated Zeb1, a key EMT-activator.

EMT process is crucial not only for physiological embryonic development, but also for pathological tissue regeneration and cancer progression, by which epithelial cells lose their polarity and acquire mesenchymal characteristics (Kalluri and Weinberg 2009). During cancer development, EMT empowers cancer cells with higher migratory/invasive and metastatic capacities. EMT is executed by a core set of transcription factors including Snail, Slug, Twist, and Zeb1. By screening the expression levels of these transcription factors on both mRNA and protein levels, we found that LINC00963 sufficiently up-regulated only Zeb1 protein level, in both nucleus and cytoplasm of LUAD cells, but not its mRNA or the expressions of other EMT transcription factors. Further analysis showed that LINC00963 suppressed the degradation of Zeb1 protein through reducing ubiquitinated level of Zeb1, suggesting that blocking the ubiquitination-mediated Zeb1 degradation is at least partially responsible for LINC00963-induced up-regulation of Zeb1. These findings naturally led to the screening of E3 ubiquitin ligases reported to regulate Zeb1 ubiquitination and our subsequent findings proved Siah1 mediated the ubiquitinated level of Zeb1 and linked Siah1 to LINC00963/HNRNPA2B1 interaction. Functionally, knocking down Zeb1 in LINC00963 overexpressed cancer cells was sufficient to abolish EMT and cell mobility, demonstrating its importance in LINC00963-induced metastatic phenotypes. This is consistent with the reported significance of Zeb1 in lung cancer (Larsen et al. 2016; Ma et al. 2019). Although we verified the regulation of LINC00963 on Zeb1 through HNRNPA2B1 and Siah1, and the functional significance of Zeb1 in mediating LINC00963-induced growth and metastasis, we noticed that knocking down Zeb1 only partially abolished LINC00963-induced malignancy, suggesting that signaling through Siah1 and Zeb1 may not be the only downstream mechanism for LINC00963. Future studies may focus on other oncogenic mechanisms of LINC00963.

In addition to firstly found that LINC00963, through interacting with HNRNPA2B1, down-regulated the stability of Siah1 mRNA, and inhibited the ubiquitinated level of Zeb1, promoted growth- and metastasis-related phenotypes of LUAD, another innovative finding from this study is that LUAD cells packaged LINC00963 in exosomes, which then induced M2 macrophage polarization. Macrophages are a critical component in tumor microenvironment and the clinical significance of M2 macrophages in promoting the progression and predicting poor prognosis of lung cancer has been well established (Mei et al. 2016; Sumitomo et al. 2019). Tumor cells employ various mechanisms to recruit and promote M2 polarization of macrophages, which in turn aggravates multiple malignant phenotypes of tumor cells (Ge and Ding 2020). Exosomes may dictate macrophage phenotypes toward either classically activated M1 or alternatively activated M2 macrophages (Baig et al. 2020). In this study, we demonstrated the presence of LINC00963 in LUAD cells-derived exosomes, the uptake of these exosomes by macrophages, and the significance of exosomal LINC00963 in inducing M2 macrophage polarization. Functionally, when co-injecting LUAD cells with macrophages pre-treated with LUAD-derived exosomes, we observed the crucial role of exosomal LINC00963 in promoting xenograft growth in vivo. Interestingly, we found that Zeb1 and CD206 expressions in these xenografts changed in the pattern as if LINC00963 was altered in LUAD cells, supporting the crosstalk between tumor cells and macrophages. The results of influence caused by macrophages pre-treated with LUAD-derived exosomes on LUAD metastatic model will strengthen our conclusion, although it is difficult for us to obtain valid results in short time, which should make intensive study in future. Also, we realized that our analysis on LUAD cells-derived exosomal LINC00963 in tumor microenvironment only focused on macrophages, ignoring other cell types such as fibroblasts, because of the important effects on EMT. Therefore, we should carefully examine the association of exosomal LINC00963 with various cell types in LUAD tissues, and comprehensively analyze its impacts on other cell types in future studies.

## Conclusion

In conclusion, this study corroborates the clinical significance and the oncogenic roles of LINC00963 in LUAD. Unlike previous works, however, this is the first study revealing two important mechanisms mediating tumor-promoting activities of LINC00963, yet independent of lncRNA/miRNA/mRNA network. Firstly, the LINC00963/HNRNPA2B1 interaction, by degrading Siah1 mRNA, suppresses ubiquitination-mediated degradation of Zeb1 and thus promotes Zeb1-regulated malignant and metastatic phenotypes. Secondly, LINC00963 is also produced by LUAD cells into exosomes, induces M2 macrophage polarization, and stimulates macrophages-induced growth of LUAD cells. Collectively and potentially with other yet-to-be-identified oncogenic mechanisms, LINC00963 stimulates the malignant progression and thus may become a promising therapeutic target of LUAD.

## Data Availability

All data generated or analysed during this study are included in this published article.

## Abbreviations

LINC00963: Long intergenic non-coding RNA 00963
LUAD: Lung adenocarcinoma
EMT: Epithelial-mesenchymal transition
lncRNAs: Long non-coding RNAs
ceRNA: Competing endogenous RNA
miRNAs: MicroRNAs
PBMCs: Peripheral blood mononuclear cells
MDMs: Monocyte-derived macrophages
M-CSF: Macrophage colony stimulating factor
FISH: Fluorescence in-situ hybridization
CHX: Cycloheximide
RIP: RNA immunoprecipitation
TEM: Transmission electron microscopy
NTA: Nanoparticle tracking analyzing
RT-qPCR: Reverse transcription followed by quantitative real-time PCR
Co-IP: Co-immunoprecipitation
HE: Hematoxylin and eosin
SD: Standard deviation
ANOVA: Analysis of variance
